# Predictive Value of Free Triiodothyronine in Patients with Acute Coronary Syndrome Undergoing Percutaneous Coronary Intervention

**DOI:** 10.31083/j.rcm2307230

**Published:** 2022-06-24

**Authors:** Zhi Qiang Yang, Xiao Teng Ma, Qiao Yu Shao, Qiu Xuan Li, Yu Fei Wang, Jing Liang, Hua Shen, Xiao Li Liu, Dong Mei Shi, Yu Jie Zhou, Zhe Fang, Zhi Jian Wang

**Affiliations:** ^1^Department of Cardiology, Beijing Anzhen Hospital, Capital Medical University, Beijing Institute of Heart Lung and Blood Vessel Disease, Beijing Key Laboratory of Precision Medicine of Coronary Atherosclerotic Disease, Clinical Center for Coronary Heart Disease, Capital Medical University, 100029 Beijing, China; ^2^Department of Cardiology, Jiangxi Provincial People's Hospital, The First Affiliated Hospital of Nanchang Medical College, 330006 Nanchang, Jiangxi, China

**Keywords:** free triiodothyronine, acute coronary syndrome, percutaneous coronary intervention, adverse cardiovascular events

## Abstract

**Background:**

Homeostasis of thyroid hormones has significant effects on the cardiovascular system. The aim of this study was to investigate the association between free triiodothyronine (FT3) and adverse cardiovascular events in patients with acute coronary syndrome (ACS) who were undergoing percutaneous coronary intervention (PCI).

**Methods:**

A total of 1701 patients with ACS undergoing PCI were included in this study. All patients were divided into three groups according to the tertiles of FT3 level: the lowest tertile (FT3 <4.51 pmol/L), the middle tertile (4.51 pmol/L ≤ FT3 < 4.89 pmol/L) and the highest tertile group (FT3 ≥4.89 pmol/L). The primary study endpoint was a composite of major adverse cardiovascular events (MACE), which included all-cause death, ischemic stroke, myocardial infarction, or unplanned repeat revascularization.

**Results:**

During a median follow-up period of 927 days, 349 patients had at least one event. Compared with patients with the highest tertile, those with the lowest tertile had a significantly higher incidence of MACE, all-cause death, MI, ischemic stroke and repeat revascularization (all *p* values < 0.05). In the multivariate Cox regression analysis, the middle tertile had similar risk of MACE (HR = 0.986, 95% CI 0.728–1.336, *p* = 0.929) as the highest tertile, but the patients with the lowest tertile had a 92.9% higher risk of MACE (HR = 1.929, 95% CI 1.467–2.535, *p *< 0.001). There was a non-linear relationship between FT3 and MACE and unplanned repeat revascularization (all *p* values for non-linear association <0.001). Adding the tertiles of FT3 level into the baseline model yielded a significant improvement in discrimination for predicting MACE (ΔAUC = 0.013, *p* = 0.025).

**Conclusions:**

A significantly reduced FT3 level was independently associated with a worse prognosis in patients with ACS undergoing PCI.

## 1. Introduction

Acute coronary syndrome (ACS), the most severe ischemic heart disease, has been recognized as one of the major causes of mortality globally and considered to be a serious public health problem. Despite the increased number of therapeutic interventions performed in patients with ACS, such as advanced pharmacotherapy and myocardial reperfusion therapy, there is still a significant incidence of major adverse cardiovascular events in these patients. Identifying the risk factors that affect the prognosis of ACS patients, is therefore, of great importance.

Homeostasis of thyroid hormones has a significant impact on the cardiovascular system [1, 2]. The abnormalities of thyroid function, subclinical or overt hypothyroidism and hyperthyroidism, have been associated with the progression of atherosclerosis and increased cardiovascular morbidity and mortality in patients with coronary artery disease (CAD) [2, 3, 4, 5, 6]. Serum triiodothyronine (T3), which is the principal bioactive thyroid hormone for cardiomyocytes, has an effect on myocardial contractility, systemic vascular resistance and cardiovascular hemodynamics. T3 works mainly through the modulation of the relative proteins coded by target genes in the cardiovascular system such as myosin heavy chains, sarcoplasmic reticulum proteins, and calcium-activated ATPase (${\mathrm{Ca}}^{2+}$-ATPase) [7]. Lower free triiodothyronine (FT3) levels are associated with a worse prognosis in acute or chronic diseases, for instance, myocardial infarction (MI) and chronic heart failure (HF) [8, 9].

Although previous studies have found that lower FT3 level have a close relationship with major adverse cardiovascular events (MACE) in ACS patients, the relationship between the level of FT3 and adverse cardiovascular events in such patients undergoing PCI has not been defined. Therefore, our study aimed to investigate the association between FT3 level and adverse cardiovascular events in patients with ACS undergoing PCI.

## 2. Materials and Methods

### 2.1 Study Population

This study is a retrospective analysis based on a single-center prospective registry (ChiCTR1800017417). From June 2016 to November 2017, 1770 ACS patients treated with PCI were admitted in our cardiovascular center and consecutively enrolled in the prospective registry. The first patient was recruited in June 2016 and follow-up was completed in December 2019. All patients were regularly followed through telephone contact by independent personnel. Four patients were lost during the period of follow-up. We ultimately included 1701 patients after excluding patients with thyroid dysfunction, infectious diseases, malignant tumors, previous coronary artery bypass grafting (CABG), and loss to follow-up.

The primary study endpoint was a composite of major adverse cardiovascular events (MACE), which consists of all-cause death, ischemic stroke, myocardial infarction, or unplanned repeat revascularization. The secondary study endpoints were each individual component of the MACE. We choose the most serious endpoint event for endpoint analysis if >1 event occurred during the follow-up (death > stroke > myocardial infarction > revascularization). If stroke, myocardial infarction or revascularization occurred more than once, the first event was selected. The endpoints were evaluated by no less than two professional cardiologists. The study was approved by the institutional review board of Beijing Anzhen Hospital, Capital Medical University and complied with the Declaration of Helsinki.

### 2.2 Demographics and Clinical Data

Data on demographics, cardiovascular risk factors, and medication history were collected by using a standard questionnaire. Coronary angiographic findings and procedural results were obtained from medical records. Body mass index (BMI) was calculated as weight (kg) divided by height (m) squared. Diabetes was diagnosed as typical symptoms of diabetes and a random plasma glucose ≥11.1 mmol/L (200 mg/dL), fasting plasma glucose (FPG) ≥7.0 mmol/L (126 mg/dL), 2-h plasma glucose ≥11.1 mmol/L (200 mg/dL) from a 75-g oral glucose tolerance test, and/or antidiabetic medication use. Blood pressure was recorded on admission, and hypertension was defined as resting blood pressure ≥140/90 mmHg, and/or antihypertension medication use. Dyslipidemia was defined as total cholesterol (TC) >5.17 mmol/L (200 mg/dL), triglycerides (TG) >1.7 mmol/L (150 mg/dL), low density lipoprotein cholesterol (LDL-C) >3.37 mmol/L (130 mg/dL), high density lipoprotein cholesterol (HDL-C) <1.03 mmol/L (40 mg/dL), and/or chronic use of lipid-lowering drugs. Peripheral arterial disease (PAD) was defined as vascular diseases correlated with the aorta and arteries except for coronary vessels confirmed by exercise-related intermittent claudication, the need for revascularization, reduced or absent peripheral pulses, angiographic stenosis of more than 50%, or combinations of these characteristics. The GRACE risk score was calculated from a range of variables including age, heart rate, systolic blood pressure (SBP), serum creatinine, heart failure (HF), cardiac arrest at admission, ST-segment deviation and elevated cardiac enzymes/markers [10].

### 2.3 Laboratory Measurements

Blood samples were collected from a cubital vein 12 hours from the time of admission in the fasting state to determine thyroid hormones, lipid profiles and other biochemical parameters, and were measured in the central laboratory of the Beijing Anzhen Hospital. Thyroid-related hormones, such as thyroid-stimulating hormone (TSH), FT3 and free thyroxine (FT4) were measured by the electrochemiluminescent immunoassay method. Serum levels of TG, TC, LDL-C and HDL-C were measured by enzymatic methods. Estimated glomerular filtration rate (eGFR) was calculated using the Chronic Kidney Disease Epidemiology Collaboration (CKD-EPI) equation. Left ventricular ejection fraction (LVEF) was measured by Doppler echocardiography by an experienced ultrasound cardiologist.

### 2.4 Statistical Analysis

Continuous variables were expressed as mean ± standard deviation (SD) or median with interquartile range (IQR). Categorical variables were presented as frequencies with percentages. All patients were divided into three groups in accordance with the tertiles of FT3 level: the lowest tertile group (FT3 <4.51 pmol/L), the middle tertile group (4.51 pmol/L ≤ FT3 < 4.89 pmol/L), and the highest tertile group (FT3 ≥4.89 pmol/L). The two independent samples *t*-test or Mann-Whitney U test was applied to compare continuous variables between groups, whereas one-way ANOVA or the Kruskal-Wallis H test was applied to compare continuous variables among multiple groups. Chi-squared test or Fisher’s exact test was applied to compare categorical variables. Receiver-operating characteristic (ROC) curve analysis was used to access the incremental effect of the tertiles of FT3 level for predicting the risk of MACE, and Delong’s test was applied to compare the area under the ROC curves (AUC) values. The linear or no-linear relationship between the FT3 level and MACE or each component of MACE was confirmed by restricted cubic spline. To further analyze the predictive value of FT3 levels, Kaplan–Meier survival curves was used to show the cumulative incidence rates of adverse cardiovascular events among groups, which were then compared with using the log-rank test. The FT3 level was analyzed as a categorical variable. We performed univariate and multivariate Cox proportional hazards regression analysis to explore the predictive value for the risk of MACE, expressed as hazard ratio (HR) and 95% confidence interval (CI). A baseline risk model was established to adjust for the potential confounders for predicting the risk of MACE.

Statistical analysis was performed with SPSS (version 24.0; IBM, IL, USA) and R Programming Language (version 4.1.0; Vienna, Austria). All probability values were 2-tailed. A *p* value of <0.05 was considered statistically significant.

## 3. Results

### 3.1 Baseline Characteristics

**Fig. 1.**
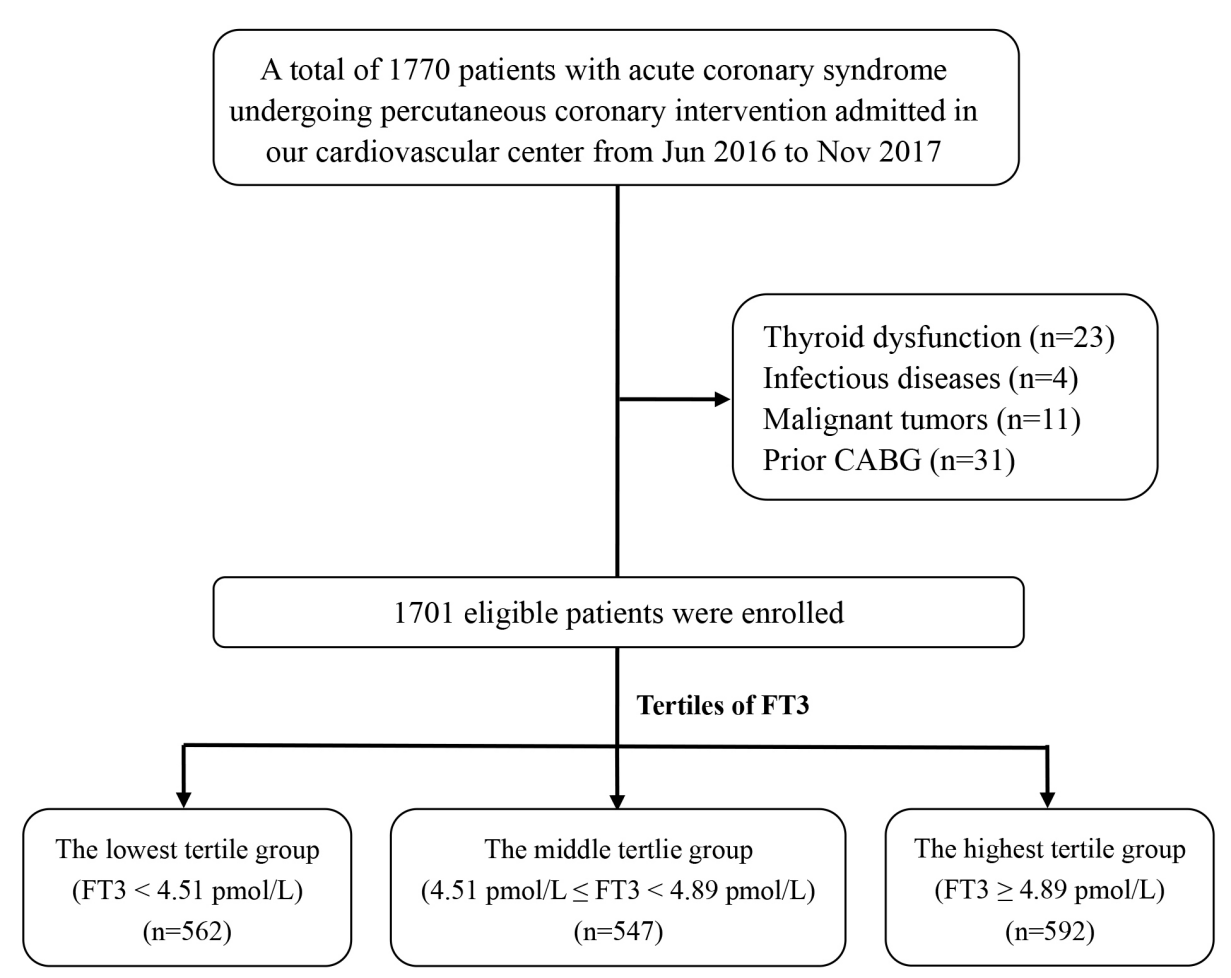
**The flow chart of the study**.

Fig. 1 shows the flow chart of the study. The average age of the study patients was 60 ± 11 years, and 77.1% were males. Table 1 demonstrates the baseline characteristics of the patient population according to the tertiles of FT3. Patients with a lower FT3 level were more likely to be male and older. Patients with lower FT3 levels had higher rates of diabetes, CKD, PAD, HF, Killip class ≥2, and ST-segment elevation myocardial infarction (STEMI), and lower rates of current smokers, unstable angina (UA), and complete revascularization. In addition, patients with lower FT3 levels had higher levels of TSH, high-sensitivity C-reactive protein (hsCRP), glycosylated hemoglobin and the GRACE risk score, but had lower LVEF, FT4, TC, and LDL-C among the 3 groups. In summary, patients with lower levels of FT3 had more baseline risk profiles.

**Table 1.** **Baseline characteristics of the patient population according to the tertiles of FT3**.

Characteristics		All patients (n = 1701)	Tertiles of FT3			
			Lowest group (n = 562)	Middle group (n = 547)	Highest group (n = 592)	*p* valve
Demographics						
	Age-years	60 ± 11	62 ± 11	59 ± 10	58 ± 10	<0.001
	Male-n (%)	1312 (77.1)	407 (72.4)	417 (76.2)	488 (82.4)	<0.001
	BMI-kg/$m^{2}$	25.7 ± 3.1	25.5 ± 3.2	25.7 ± 3.1	25.9 ± 3.0	0.051
Risk factors						
	Current smokers-n (%)	756 (44.4)	219 (39.0)	239 (43.7)	298 (50.3)	<0.001
	Family history of CAD-n (%)	546 (32.1)	177 (31.5)	174 (31.8)	195 (32.9)	0.858
	Hypertension-n (%)	1080 (63.5)	368 (64.8)	358 (65.4)	358 (60.5)	0.163
	Diabetes-n (%)	787 (46.3)	281 (50.0)	259 (47.3)	247 (41.7)	0.016
	Dyslipidemia-n (%)	1366 (80.3)	453 (80.6)	438 (80.1)	475 (80.2)	0.974
	CKD-n (%)	52 (3.1)	33 (5.9)	12 (2.2)	7 (1.2)	<0.001
	Previous MI-n (%)	325 (19.1)	125 (22.2)	101 (18.5)	99 (16.7)	0.052
	Past PCI-n (%)	338 (19.9)	119 (21.2)	109 (19.9)	110 (18.6)	0.544
	PAD-n (%)	177 (10.4)	78 (13.9)	61 (11.2)	38 (6.4)	<0.001
	Heart Failure-n (%)	118 (6.9)	72 (12.8)	31 (5.7)	15 (2.5)	<0.001
	LVEF (%)	63 ± 8	62 ± 9	63 ± 8	64 ± 6	0.002
	Killip class ≥2-n (%)	63 (3.7)	40 (7.1)	18 (3.3)	5 (0.8)	<0.001
	SBP on admission (mmHg)	130 ± 17	130 ± 17	130 ± 17	129 ± 16	0.562
	HR on admission (bmp)	69 ± 9	69 ± 10	69 ± 10	69 ± 9	0.979
	GRACE risk score	104 ± 39	113 ± 43	103 ± 38	96 ± 34	<0.001
Type of ACS						
	UA-n (%)	1261 (74.1)	401 (71.4)	399 (72.9)	461 (77.9)	0.030
	NSTEMI-n (%)	222 (13.1)	74 (13.2)	76 (13.9)	72 (12.2)	0.683
	STEMI-n (%)	218 (12.8)	87 (15.5)	72 (13.2)	59 (10.0)	0.019
Laboratory measurements						
	TSH (mIU/L)	2.08 ± 0.99	2.24 ± 1.02	2.08 ± 0.94	1.94 ± 0.98	<0.001
	FT4 (pmol/L)	11.37 ± 1.53	11.00 ± 1.55	11.52 ± 1.33	11.59 ± 1.61	<0.001
	hsCRP (mmol/L)	1.36 (0.65–3.49)	1.68 (0.72–5.05)	1.34 (0.67–3.40)	1.21 (0.55–2.63)	<0.001
	TG (mmol/L)	1.45 (1.01–2.07)	1.43 (0.98–2.01)	1.43 (1.03–2.10)	1.50 (1.03–2.10)	0.364
	TC (mmol/L)	4.15 ± 1.00	4.11 ± 1.00	4.10 ± 1.00	4.24 ± 0.99	0.028
	HDL-C (mmol/L)	1.03 ± 0.23	1.02 ± 0.25	1.02 ± 0.23	1.04 ± 0.23	0.078
	LDL-C (mmol/L)	2.45 ± 0.81	2.42 ± 0.80	2.40 ± 0.80	2.51 ± 0.82	0.020
	FPG (mmol/L)	5.81 (5.23–6.94)	5.89 (5.26–7.14)	5.78 (5.24–6.83)	5.73 (5.19–6.85)	0.104
	Glycosylated hemoglobin (%)	6.1 (5.6–7.1)	6.2 (5.7–7.2)	6.1 (5.5–7.1)	6.0 (5.5–7.0)	0.011
Angiographic findings						
	LM/three-vessel disease-n (%)	81 (4.8)	21 (3.7)	30 (5.5)	30 (5.1)	0.358
	Two-vessel disease-n (%)	488 (28.7)	140 (24.9)	167 (30.5)	181 (30.6)	0.054
	One-vessel disease-n (%)	260 (15.3)	79 (14.1)	86 (15.7)	95 (16.0)	0.606
	Proximal LAD stenosis-n (%)	857 (50.4)	286 (50.9)	272 (49.7)	299 (50.5)	0.925
	SYNTAX score	21.3 ± 11.0	21.7 ± 10.6	20.9 ± 11.0	21.1 ± 11.2	0.245
Procedural results						
	DES-n (%)	1398 (82.2)	455 (81.0)	447 (81.7)	496 (83.8)	0.430
	BRS-n (%)	94 (5.5)	36 (6.4)	33 (6.0)	25 (4.2)	0.220
	Complete revascularization-n (%)	1048 (61.6)	318 (56.6)	349 (63.8)	381 (64.4)	0.011
Medication at discharge						
	Aspirin-n (%)	1685 (99.1)	552 (98.2)	543 (99.3)	590 (99.7)	0.033
	Clopidogrel-n (%)	1563 (91.9)	526 (93.6)	507 (92.7)	530 (89.5)	0.029
	Ticagrelor-n (%)	138 (8.1)	36 (6.4)	40 (7.3)	62 (10.5)	0.029
	Statins-n (%)	1701 (100.0)	571 (100.0)	571 (100.0)	559 (100.0)	
	ACEI/ARBs-n (%)	822 (48.3)	299 (53.2)	272 (49.7)	251 (42.4)	0.001
	Beta-blockers-n (%)	1193 (70.1)	407 (72.4)	371 (67.8)	415 (70.1)	0.247

The groups were stratified by the tertiles of FT3.

### 3.2 Clinical Outcomes and Kaplan-Meier Analysis

During the 927-day (IQR, 927–1109 days) follow-up period, 349 (20.5%) patients with ACS undergoing PCI developed MACE, which included 44 (2.6%) all-cause death, 22 (2.6%) ischemic stroke, 41 (2.4%) MI, and 242 (14.2%) unplanned repeat revascularization. The incidence of adverse events was compared among groups divided by the tertiles of the FT3 level. Patients in lowest level of FT3 had a significantly higher incidence of MACE than the highest and middle groups (31.5% versus 15.0% and 15.2%; chi-square *p *< 0.001). Compared with the highest FT3 level, the rate of all-cause death, ischemic stroke, MI and unplanned repeat revascularization increased significantly in patients with lower FT3 levels (all chi-square *p *< 0.01) (Table 2).

**Table 2.** **Clinical outcomes in patients based on tertiles of FT3**.

Cardiovascular outcomes	Lowest group (n = 562)	Middle group (n = 547)	Highest group (n = 592)	*p* valve
MACE	177 (31.5%)	83 (15.2%)	89 (15.0%)	<0.001
All-cause death	33 (5.9%)	5 (0.9%)	6 (1.0%)	<0.001
Nonfatal stroke	14 (2.5%)	4 (0.7%)	4 (0.7%)	0.009
Nonfatal MI	24 (4.3%)	7 (1.3%)	10 (1.7%)	0.002
Unplanned repeat revascularization	106 (18.9%)	67 (12.2%)	69 (11.7%)	0.001

The groups were stratified by the tertiles of FT3.

**Fig. 2.**
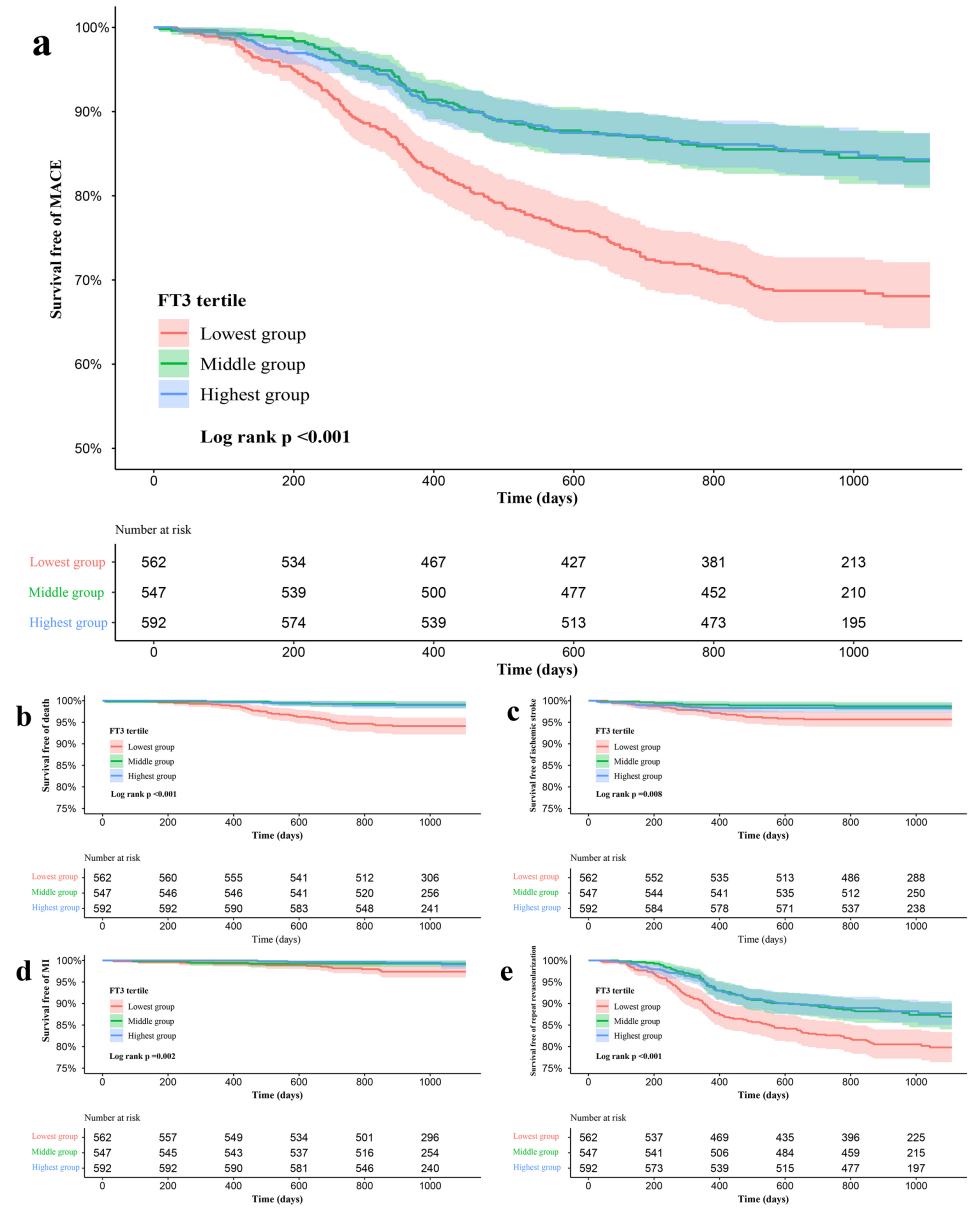
**Kaplan-Meier curves for no events survival according to the tertiles of FT3**. (a) Kaplan-Meier curves for MACE. (b) Kaplan-Meier curves for All-cause death. (c) Kaplan-Meier curves for ischemic stroke. (d) Kaplan-Meier curves for MI. (e) Kaplan-Meier curves for Unplanned repeat revascularization.

Kaplan-Meier curves for survival free of any MACE and its individual components according to the tertiles of FT3 levels are shown in Fig. 2. The survival free of MACE in the lowest FT3 tertile group was significantly lower than that in the highest and middle groups at follow-up (Fig. 2a, Log-rank *p *< 0.001), and the difference was significant in each component of MACE (Fig. 2b–e, all Log-rank *p *< 0.01).

### 3.3 Cox Regression Analysis for MACE 

In Cox regression analysis, compared with the highest tertile, the middle tertile had a similar risk of MACE (HR = 0.986, 95% CI 0.728–1.336), but patients with the lowest tertile had a 92.9% higher risk of MACE (HR = 1.929, 95% CI 1.467–2.535, *p *< 0.001) (Table 3).

**Table 3.** **Cox regression analysis for MACE**.

Variables			Univariate analysis		Multivariate analysis	
			HR (95% CI)	*p* value	HR (95% CI)	*p* value
MACE						
	FT3 tertiles					
		Highest group	Ref		Ref	
		Middle group	0.999 (0.741–1.348)	0.997	0.986 (0.728–1.336)	0.929
		Lowest group	2.275 (1.763–2.935)	<0.001	1.929 (1.467–2.535)	<0.001
	Age		1.007 (0.997–1.017)	0.172	0.995 (0.977–1.013)	0.589
	Sex		1.036 (0.804–1.334)	0.784	0.874 (0.640–1.194)	0.397
	Current smoking		1.144 (0.927–1.411)	0.210	1.248 (0.970–1.605)	0.085
	Hypertension		1.065 (0.855–1.326)	0.576	1.172 (0.907–1.515)	0.224
	Diabetes		1.549 (1.254–1.913)	<0.001	1.373 (1.099–1.716)	0.005
	Dyslipidemia		1.333 (1.002–1.774)	0.049	0.758 (0.539–1.067)	0.112
	HF		1.903 (1.369–2.645)	<0.001	1.438 (0.912–2.267)	0.118
	CKD		2.729 (1.771–4.204)	<0.001	1.841 (1.143–2.967)	0.012
	Previous MI		1.507 (1.186–1.915)	<0.001	0.935 (0.697–1.253)	0.651
	Past PCI		1.553 (1.227–1.966)	<0.001	1.608 (1.213–2.132)	0.001
	Types of ACS					
		UA	Ref		Ref	
		NSTEMI	1.160 (0.857–1.571)	0.337	0.977 (0.597–1.599)	0.926
		STEMI	1.025 (0.747–1.408)	0.877	0.964 (0.456–2.036)	0.923
	GRACE risk score		1.003 (1.000–1.005)	0.052	0.999 (0.991–1.006)	0.711
	TG		1.110 (1.051–1.173)	<0.001	1.049 (0.980–1.121)	0.166
	HDL-C		0.379 (0.235–0.611)	<0.001	0.438 (0.237–0.810)	0.009
	LDL-C		1.185 (1.049–1.338)	0.006	1.229 (1.070–1.411)	0.004
	hsCRP		1.034 (1.019–1.049)	<0.001	1.019 (1.001–1.037)	0.041
	TSH		1.187 (1.078–1.307)	<0.001	1.104 (0.995–1.226)	0.063
	FT4		0.861 (0.801–0.924)	<0.001	0.889 (0.824–0.960)	0.003
	Complete revascularization		0.416 (0.337–0.515)	<0.001	0.576 (0.453–0.733)	<0.001
	SYNTAX score		1.035 (1.026–1.044)	<0.001	1.025 (1.014–1.037)	<0.001
	Medication at discharge					
		Aspirin	0.242 (0.129–0.454)	<0.001	0.454 (0.233–0.882)	0.020
		ACEI/ARBs	1.139 (0.923–1.405)	0.224	0.911 (0.716–1.158)	0.444
		Beta-Blockers	0.769 (0.617–0.958)	0.019	0.653 (0.519–0.822)	<0.001

The groups were stratified by the tertiles of FT3.

**Fig. 3.**
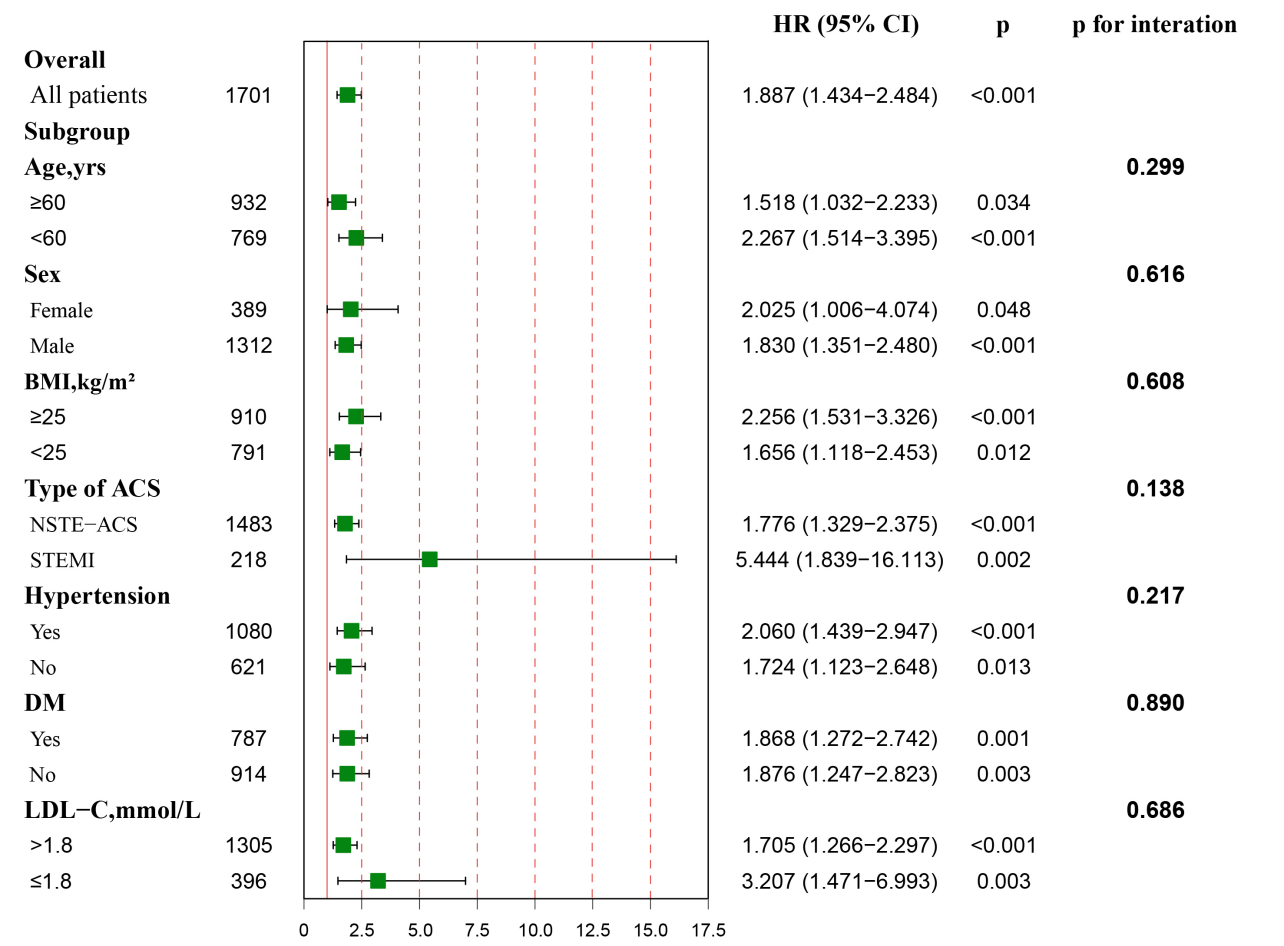
**Association between FT3 levels and the risk of MACE in overall and subgroups**. Hazard ratio (HR) was calculated by multivariate Cox regression analysis. The analysis was performed after adjusting for variates including age, gender, BMI, current smoking, hypertension, DM, dyslipidemia, HF, CKD, previous MI, past PCI, Type of ACS, GRACE risk score, TG, HDL-C, LDL-C, hs-CRP, TSH, FT4, complete revascularization, SYNTAX score, medication at discharge (aspirin, ACEI/ARBs, Beta-blockers). Red vertical solid line represents the HR value of 1.

A subgroup analysis was performed to further estimate the risk stratification value of FT3 level for MACE in the study population. The association between decreased FT3 level and higher risk of MACE was consistent between several patient characteristics including sex [HR (95% CI), 2.025 (1.006–4.074) Female, *p* = 0.048 vs. 1.830 (1.351–2.480) Male, *p *< 0.001], age [HR (95% CI), 2.267 (1.514–3.395) for younger, *p *< 0.001 vs. 1.518 (1.032–2.233) for elder, *p* = 0.034], weight [HR (95% CI), 1.656 (1.118–2.453) for normal weight, *p* = 0.012 vs. 2.256 (1.531–3.326) for overweight, *p *< 0.001], different types of ACS [HR (95% CI), 1.776 (1.329–2.375) for NSTE-ACS, *p *< 0.001 vs. 5.444 (1.839–16.113) for STEMI, *p* = 0.002], hypertension [HR (95% CI), 2.060 (1.439–2.947) for patient with hypertension, *p *< 0.001 vs. 1.724 (1.123–2.648) for patient without hypertension, *p* = 0.013], DM [HR (95% CI), 1.868 (1.272–2.742) for patient with DM, *p* = 0.001 vs. 1.876 (1.247–2.823) for patient without DM, *p* = 0.003], LDL-C [HR (95% CI), 1.705 (1.266–2.297) for patient with LDL-C >1.8 mmol/L, *p *< 0.001 vs. 3.207 (1.471–6.993) for patient with LDL-C ≤1.8 mmol/L, *p* = 0.003] (all *p* values for interaction >0.05) (Fig. 3).

### 3.4 The Relationship between the FT3 Levels and MACE and Each Component of MACE

**Fig. 4.**
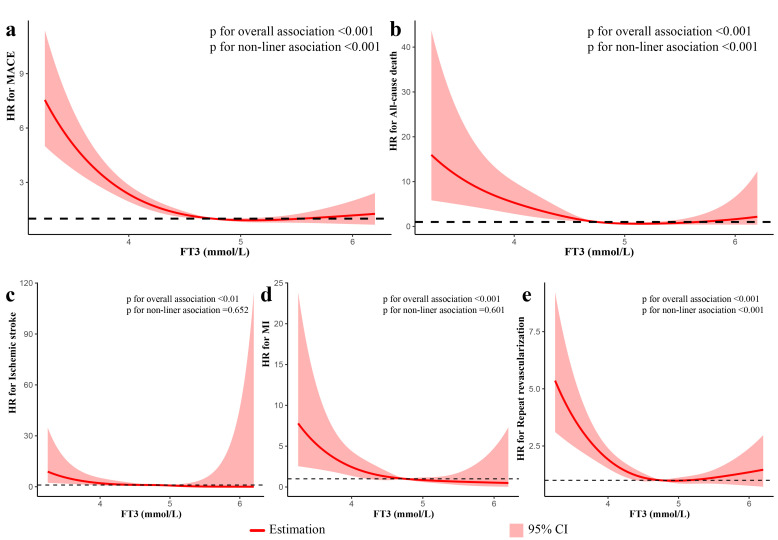
**Restricted spline curves for the associations of FT3 with MACE and each component of MACE in patients with ACS undergoing PCI in univariate analysis**. The red lines represent the hazard ratio, and the shaded area represents the 95% confidence intervals (CI). (a) Association of FT3 with MACE. (b) Association of FT3 with All-cause death. (c) Association of FT3 with ischemic stroke. (d) Association of FT3 with MI. (e) Association of FT3 with Unplanned repeat revascularization.

**Fig. 5.**
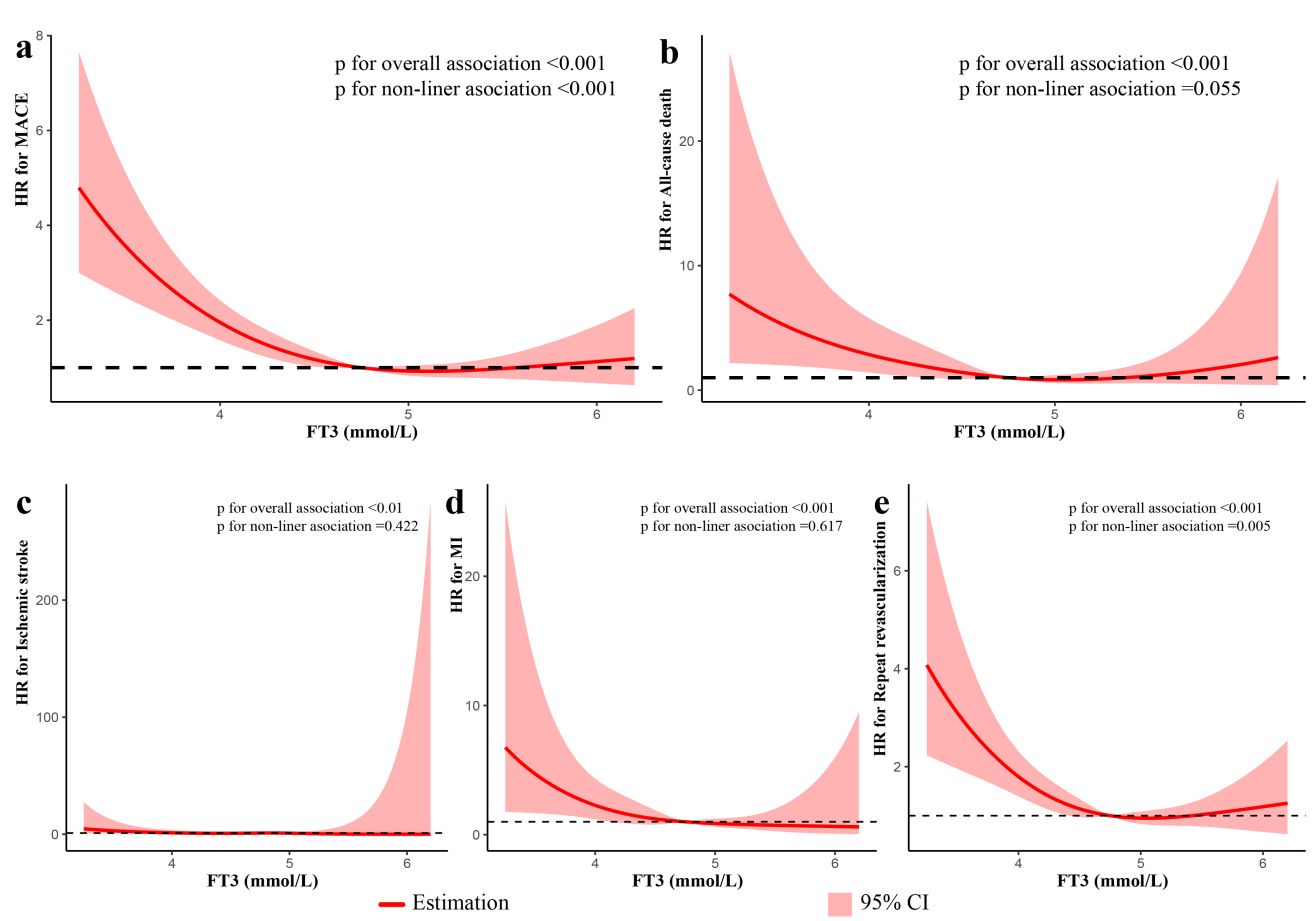
**Restricted spline curves for the associations of FT3 with MACE and each component of MACE in patients with ACS undergoing PCI in multivariate analysis**. The analysis was performed after adjusting for variates including age, gender, BMI, current smoking, hypertension, DM, dyslipidemia, HF, CKD, previous MI, past PCI, Type of ACS, GRACE risk score, TG, HDL-C, LDL-C, hs-CRP, TSH, FT4, complete revascularization, SYNTAX score, medication at discharge (aspirin, ACEI/ARBs, Beta-blockers). The red lines represent the hazard ratio, and the shaded area represents the 95% confidence intervals (CI). (a) Association of FT3 with MACE. (b) Association of FT3 with All-cause death. (c) Association of FT3 with ischemic stroke. (d) Association of FT3 with MI. (e) Association of FT3 with Unplanned repeat revascularization.

Restricted cubic splines were used to investigate the relationships between FT3 and MACE and each component of MACE. Fig. 4 showed a non-linear association between FT3 and MACE, all-cause death and unplanned repeat revascularization (all *p* values for non-linear association <0.001), while FT3 level has a linear relationship with ischemic stroke and MI. In multivariable analysis, there was a non-linear association between FT3 and MACE and unplanned repeat revascularization (all *p* values for non-linear association <0.01), while FT3 level has a linear relationship with all-cause death, ischemic stroke and MI (Fig. 5).

### 3.5 Incremental Value of FT3 Level for Predicting MACE

**Fig. 6.**
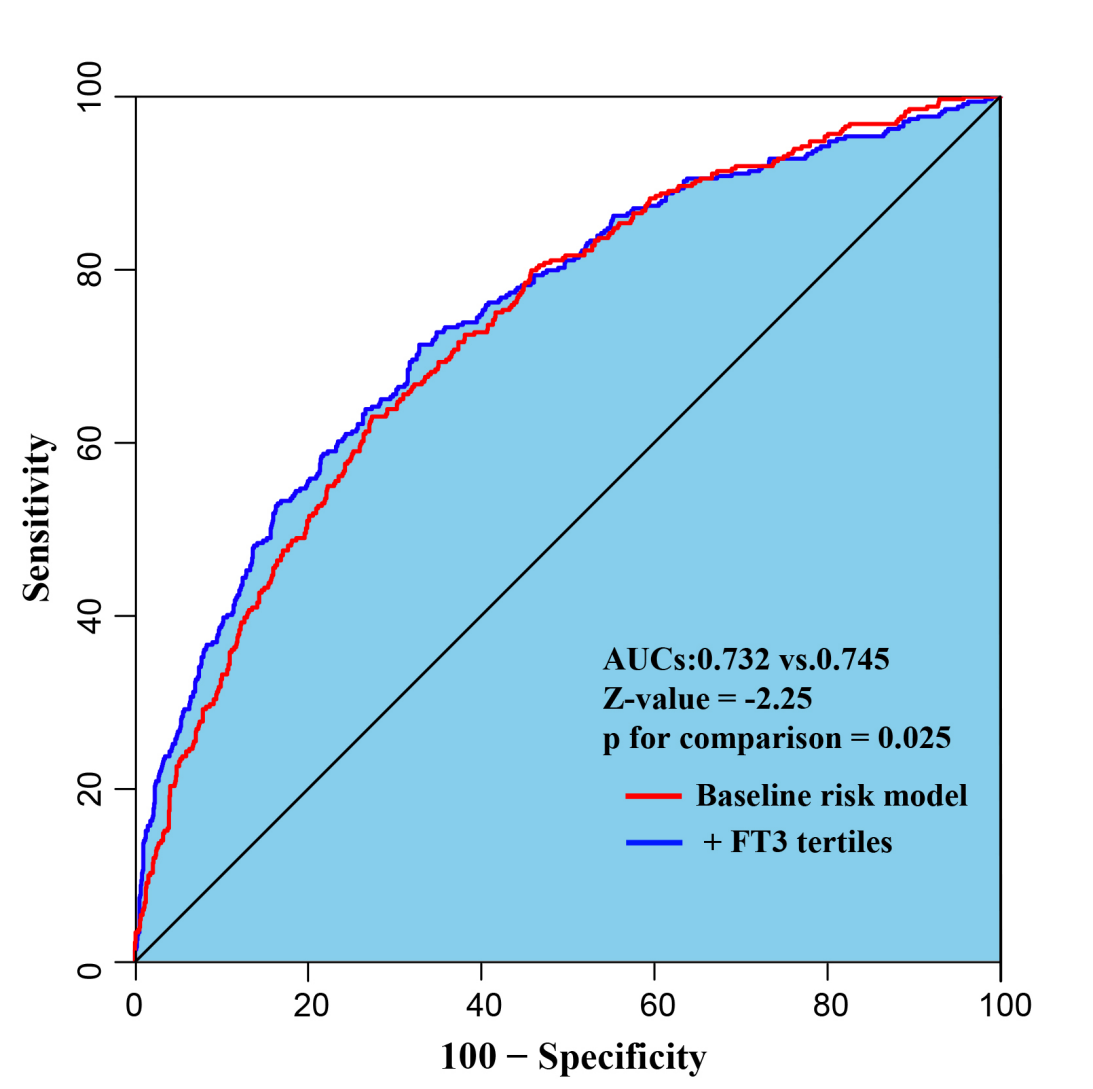
**Receiver operating characteristic curves evaluating incremental effect of FT3 beyond baseline risk model**. The baseline risk model includes age, sex, current smoking, hypertension, diabetes, dyslipidemia, HF, CKD, previous MI, past PCI, types of ACS, GRACE risk score, TG, HDL-C, LDL-C, hs-CRP, hTSH, FT4, complete revascularization, SYNTAX score, medication at discharge (aspirin, ACEI/ARB, Beta-Blockers).

The baseline risk model included risk factors such as age, sex, current smoking, hypertension, diabetes, dyslipidemia, HF, CKD, previous MI, past PCI, types of ACS, GRACE risk score, TG, HDL-C, LDL-C, hs-CRP, hTSH, FT4, complete revascularization, SYNTAX score, and medication at discharge (aspirin, ACEI/ARB, Beta-Blockers). The addition of the tertiles of FT3 level has a significant incremental effect on baseline tables for predicting the risk of MACE (AUC: baseline risk model, 0.732 vs. baseline risk model + FT3 level, 0.745, ΔAUC = 0.013, *p* for comparison = 0.025 by Delong’s test) (Fig. 6).

## 4. Discussion

The present study retrospectively investigated the predictive value of FT3 levels for adverse cardiovascular events in ACS patients undergoing PCI. The major findings are: (1) compared with the highest level of FT3, those with lower FT3 levels had a significantly higher incidence of MACE; (2) decreased FT3 levels were an independent predictor of poor prognosis; (3) there was a non-linear association between FT3 level and MACE, and unplanned repeat revascularization, but not with all cause death, ischemic stroke and MI; (4) the additional prognosis value of the baseline risk model was found after adding the tertiles of each FT3 level.

Thyroid hormone has a direct effect on the cardiac, and is an important regulator of cardiac function and hemodynamics [2]. Thyroid hormones affect cardiac status: (1) by direct genomic actions on cardiomyocytes via binding to nuclear receptors, which regulates the expression of target genes encoding sodium/potassiumtransporting ATPases, α-myosin heavy chain and sarcoplasmic/endoplasmic reticulum calcium ATPase 2 (SERCA2), and negatively regulates the transcription of β-myosin heavy chain and phospholamban (PLN); (2) by non-genomic actions on the ion channels for sodium, potassium and calcium and influence various intracellular pathways of cardiomyocytes and vascular smooth-muscle cells, which determines cardiovascular haemodynamics, and cardiac contractility [7, 11, 12, 13, 14]. T3 and T4 are the two main iodinated hormones that are secreted by thyroid gland, and both can generate biological effects by combining the thyroid hormone receptors. There is a tenfold greater affinity of the thyroid hormone receptors for T3 compared to T4 [2, 15]. Therefore, T3 is universally regarded as the biologically active form of thyroid hormone.

Thyroid hormone metabolism disorders increase the risk of CAD and cardiovascular death, therefore evaluation of FT3 levels may benefit to identify patients who are susceptible to cardiovascular disease [16, 17, 18]. Therefore, assessment of FT3 levels has important clinical significance for risk stratification and individualized treatment of such patients.

Previous studies have found that patients with overt hyperthyroidism present with hyperdynamic circulation, such as increased cardiac preload and contractility, decreased systemic vascular resistance, increased heart rate, increased systolic blood pressure and pulmonary hypertension, while overt hypothyroidism is associated with a reduction in cardiac output and cardiac contractility, decreased heart rate, increased peripheral vascular resistance, dyslipidemia, and atherosclerotic plaque development and instability [1, 4, 5, 11]. Both overt hyperthyroidism and hypothyroidism are significantly associated with a higher incidence of adverse cardiovascular events [19, 20, 21, 22]. Even CAD patients with mild thyroid dysfunction and subclinical hyperthyroidism may also experience adverse cardiovascular events, such as supraventricular tachyarrhythmias, especially atrial fibrillation [23], and subclinical hypothyroidism is a strong indicator for the risk of atherosclerosis and myocardial infarction [1, 24, 25], both of which may markedly increase cardiovascular morbidity and mortality.

Several studies have shown a significant correlation between FT3 levels and adverse cardiovascular events. Lower FT3 levels have been correlated with HF, lower LVEF and serum biomarkers of myocardial injury, such as troponin T (cTnT) and N-terminal pro-brain natriuretic peptide (NT-proBNP) [26, 27]. Lymvaios *et al*. [28] found a significant correlation of low T3 with impaired ventricular function in patients with acute MI (AMI), and that T3 levels appear to be an independent predictor of late functional recovery. In addition, D. H. Kim *et al*. [29] reported that in STEMI patients, the degree of transmural involvement accessed by contrast-enhanced cardiac magnetic resonance (CMR) imaging is strongly correlated with T3 levels. Chang *et al*. [30] demonstrated that low FT3 levels was an independent indicator of long-term worse prognosis in STEMI patients undergoing PCI. Other studies also found that low T3 levels have been correlated with short and long-term mortality in STEMI patients undergoing PCI [9, 31]. Similar to patients with STEMI, NSTE-ACS patients with low FT3 levels had an increased risk of mortality during 1-month and 1-year follow-up [17]. Furthermore, reverse T3 (rT3) was independently associated with 1-year mortality [32]. In most of the above studies, PCI was not performed in all the study populations; therefore, whether PCI combined with the FT3 level improves the prognostic value of the baseline risk factors is controversial. Our study is unique in that all patients received PCI, and therefore allowed us to assess the prognostic value of the FT3 level and its incremental effect on risk stratification based on traditional risk factors in these patients.

In the study, we found that low FT3 has an impact on cardiovascular system and clinical outcomes. However, there are a large number of factors contributing to low FT3, such as demographic factors (age, gender and BMI [33, 34, 35, 36]), lifestyle factors (alcohol consumption [37]), diet (soy-based food [38, 39], olive oil [40]), exercise [41, 42], pollutants (chemicals and heavy metals) [43]. We also found that age, gender and BMI, which were all associated with low FT3, were significantly different from MACE. Besides, after multivariate analysis, decreased FT3 levels were an independent predictor of poor prognosis.

Rapid down-regulation of the thyroid hormone system in patients with acute myocardial ischemia might be of great help to reducing oxygen demands on the myocardium. Several studies confirmed that the function of pituitary gland and the release of TSH could directly affected by cytokines, such as Interleukin-6 (IL-6), tumor necrosis factor-α (TNF-α), and interferon-γ (INF-γ) [44, 45, 46]. L. Friberg, S *et al*. [47] prospectively revealed that patients with angina had lower T3 levels, smaller infarctions, and higher levels of C-reactive protein and interleukin 6 (IL-6). The low T3 syndrome might be a beneficial and physiological adaptation to early stress response of the ACS [48, 49]. However, this view has been questioned in the past several years, and low T3 syndrome may not only be an adaptive response to cardiovascular diseases [50]. There are several possible mechanisms. First, T3 was the major thyroid hormone. Low T3 or FT3 in patients with low T3 syndrome can lead to the progression of cardiovascular diseases, worsen cardiac structure and function, and increased the risk of mortality [46, 51]. Second, low T3 syndrome remains an independent prognostic factor in cardiovascular patients even after the adjustment of traditional risk factors. These results indicate that there is a direct link between low T3 syndrome and mortality risk in these patients rather than an adaptive response [52]. Third, clinical and experimental studies have reported that in cardiovascular patients or animal models, T3 treatment can produce a beneficial effect on cardiac function [53, 54]. Finally, previous studies revealed that decreased thyroid levels might have existed before the infarction occurred [47]. This suggests that there is a relationship between the alteration of T3 levels and cardiovascular diseases. Lower T3 levels depress cardiac function, and cardiovascular diseases may result in lower T3 levels. As a result, a persistent down-regulation of thyroid function might be maladaptive in cardiovascular patients. Several studies have shown that ACS patients diagnosed with the low T3 syndrome have poorer cardiovascular outcomes [31, 55, 56].

Previous studies have demonstrated that lower FT3 levels have adverse prognostic value for ACS patients undergoing PCI. Therefore, incorporating assessment and intervention of thyroid function into long-term management might be beneficial to these patients. F. Forini *et al*. [54] found that long-term L-T3 replacement in a rat model after MI reduces the infarct size by 50% and prevents the progression to HF. K. K. Henderson *et al*. [57] revealed that T3 treatment to euthyroid levels improves systolic function, and tends to improve diastolic function in an animal model of myocardial infarction-induced HF. In clinical studies, J. D. Klemperer *et al*. [58] found that raising serum T3 concentration in patients undergoing coronary artery bypass surgery improves cardiac function, but there was no significant difference in outcomes. A double-blind, randomized, placebo-controlled trial found that treatment with T3 in children after cardiopulmonary bypass operation improves myocardial function [53]. A recent RCT found that compared with placebo, patients with subclinical hypothyroidism and acute MI treated with Levothyroxine did not improve LVEF during a 52-week follow-up period [59]. The therapeutic mechanisms of action of TH are not well studied in humans, but *in vitro* and *in vivo* disease models have provided important knowledge of its repair and regeneration properties. The TH signaling pathway is a universally conserved pathway with pleiotropic effects that regulates biological development, metabolism and homeostasis, as well as having an important effect in tissue repair/regeneration. Indeed, a growing number of experimental and clinical studies suggest that TH may be critical for recovery after injury. Katzeff H L *et al*. [60] revealed that T3 can activate Akt signaling in rat cardiomyocytes, which protects myocytes against serum starvation-induced cell death, and also found that T3 supplementation protected myocytes against ischemic-induced apoptosis, which may be mediated by Akt signaling [61]. In an ischemia/reperfusion model, Pantos *et al*. [62] demonstrated that T3 administration had an anti-apoptotic effect associated with lower levels of p38 mitogen-activated protein kinase (MAPK). Several studies illustrated that L-T3 therapy increases the expression of hypoxia-inducible factor-1α (HIF-1α) and mitochondrial transcription factor A (mt-TFA), which have an important role in maintaining cell survival during myocardial ischemia, attenuating left ventricular remodeling and preserving post-MI cardiac performance [63, 64, 65]. Inhibits the expression of thyroid hormone receptors (TRs) can activate to normal and facilitate the adult transcriptional phenotype under pathological conditions by administrating TH, which might be relevant to therapeutic interventions [66, 67]. In addition, TH appears to regulate the differentiation of cardiac cells (e.g., regulation of contractile proteins and protein kinases, cytoskeletal orientation and cell geometry) and the cellular response to stress [68, 69, 70]. Whether T3 supplementation can benefit patients with ACS undergoing PCI and the therapeutic mechanism of TH are still unknown. The Thyroid Hormone Replacement therapy in ST elevation myocardial infarction (THiRST) trial, an ongoing RCT, was designed to further evaluate the effect of T3 treatment in patients with AMI [65], and the results of this study are noteworthy. Future RCTs will be designed to further evaluate the effect of T3 treatment in patients with ACS.

### Limitations 

This study retrospectively demonstrated the predictive value of FT3 levels for adverse cardiovascular events in ACS patients undergoing PCI, and demonstrated that FT3 levels are a useful predictor in clinical practice and have a positive impact on the traditional risk factors-based risk stratification. There are several limitations. (1) This was a single-center, observational study with strict exclusion criteria, and a relatively small number of patients. Some key data were collected from medical records, so the potential confounders and selections bias could not be completely adjusted, which might limit the application of our results. (2) The FT3 level was measured only once in the present study and the alteration of FT3 levels during follow-up were not known, which may have a greater predictive value for a poor prognosis. (3) Our study does not include rT3 and cytokines, which may diminish its potential correlations. (4) We only analyzed the correlation between FT3 levels and adverse cardiovascular events; other related indicators such as TT3, TT4, FT4, rT3, and the FT3/FT4 ratio might also have some potential influence for cardiovascular events. (5) There are many factors associated with low FT3, we just analyzed a few of them in this observational study.

## 5. Conclusions

In ACS patients undergoing PCI, the present study revealed that decreased FT3 levels was significantly associated with a worse prognosis. There was a non-linear association between FT3 and MACE, all-cause death and unplanned repeat revascularization. The addition of FT3 levels to the baseline risk model significantly improved the ability for risk prediction. Additional large scale randomized studies are needed to confirm whether FT3 levels have a positive effect on improving clinical outcomes.

## Abbreviations

FT3, free triiodothyronine; BMI, body mass index; CAD, coronary artery disease; CKD, chronic kidney disease; MI, myocardial infarction; PCI, percutaneous coronary intervention; PAD, peripheral artery disease; LVEF, left ventricular ejection fraction; SBP, systolic blood pressure; HR, heart rate; GRACE, global registry of acute coronary events; ACS, acute coronary syndrome; UA, unstable angina pectoris; MI, myocardial infraction; NSTEMI, non-ST segment elevation myocardial infarction; STEMI, ST segment elevation myocardial infarction; TSH, thyroid stimulating hormone; FT4, free thyroxine; hsCRP, high-sensitivity C-reactive protein; TG, triglycerides; TC, total cholesterol; HDL-C, high-density lipoprotein-cholesterol; LDL-C, low-density lipoprotein-cholesterol; FPG, fasting plasma glucose; LM, left-main; LAD, left anterior descending; SYNTAX, SYNergy between percutaneous coronary intervention with TAXus and cardiac surgery; DES, drug eluting stent; BRS, bioresorbable scaffold; ACEI, angiotensin converting enzyme inhibitor; ARB, angiotensin II receptor blocker; MACE, major adverse cardiovascular events; eGFR, estimated glomerular filtration rate; HR, hazard ratio; CI, confidence interval; Ref, reference.
